# Resilience of spider communities affected by a range of silvicultural treatments in a temperate deciduous forest stand

**DOI:** 10.1038/s41598-021-99884-8

**Published:** 2021-10-15

**Authors:** Ferenc Samu, Zoltán Elek, Bence Kovács, Dávid Fülöp, Erika Botos, Dénes Schmera, Réka Aszalós, András Bidló, Csaba Németh, Vivien Sass, Flóra Tinya, Péter Ódor

**Affiliations:** 1 Centre for Agricultural Research, Plant Protection Institute, Eötvös Loránd Research Network, Herman Ottó út 15, Budapest, 1022 Hungary; 2 MTA-ELTE-MTM Ecology Research Group, Pázmány P. stny. 1/C, Budapest, 1117 Hungary; 3 Centre for Ecological Research, Institute of Ecology and Botany, Alkotmány u. 2-4, Vácrátót, 2163 Hungary; 4 Balaton Limnological Research Institute, Eötvös Loránd Research Network, Klebelsberg Kuno u. 3, Tihany, 8237 Hungary; 5 Faculty of Forestry, Institute of Environmental and Earth Sciences, University of Sopron, Bajcsy-Zsilinszky u. 4, Sopron, 9400 Hungary

**Keywords:** Ecology, Zoology

## Abstract

To secure the ecosystem services forests provide, it is important to understand how different management practices impact various components of these ecosystems. We aimed to uncover how silvicultural treatments affected the ground-dwelling spider communities during the first five years of a forest ecological experiment. In an oak-hornbeam forest stand, five treatments, belonging to clear-cutting, shelterwood and continuous cover forestry systems, were implemented using randomised complete block design. Spiders were sampled by pitfall traps, and detailed vegetation, soil and microclimate data were collected throughout the experiment. In the treatment plots spider abundance and species richness increased marginally. Species composition changes were more pronounced and treatment specific, initially diverging from the control plots, but becoming more similar again by the fifth year. These changes were correlated mostly to treatment-related light intensity and humidity gradients. The patchy implementation of the treatments induced modest increase in both gamma and beta diversity of spiders in the stand. Overall, spiders gave a prompt and species specific response to treatments that was by the fifth year showing signs of relatively quick recovery to pre-treatment state. At the present fine scale of implementation the magnitude of changes was not different among forestry treatments, irrespective of their severity.

## Introduction

Deciduous forests play an important role in the assimilation of carbon dioxide, in carbon storage, in reducing air pollution and flood risks. They also provide timber as raw material and are essential for human wellbeing^1,2^. The sustainable functioning of forests largely depends on the ecological stability and resilience^3^ of their species assemblages and the ecological links between them. Since nearly all temperate forests are under forestry management^4^, the functioning of forest ecosystems largely depends on the interaction between the ecological processes and human management. To secure the ecosystem services forests provide, it is of paramount importance to understand how management practices impact different components of the system.

The most widespread forest management type in European broadleaved forests in the past two centuries is rotation forestry, including uniform shelterwood and clear-cutting silvicultural systems. These practices eventually lead to even-aged forest stands^5,6^. Rotation forestry has many negative long-term effects on habitat preservation, forest health and biodiversity^7^. The emerging need for healthy and productive forests, which also deliver the conservation of biodiversity, has led to modifications of basic even-aged rotational practices. These included leaving retention tree groups in the clear-cut areas^8^, or various implementations of the shelterwood system^9^. Continuous cover forestry is both a traditional and a new forestry practice. On the one hand, this management type has a long tradition in several European countries. However, with rotation forestry becoming the norm, it became less-and-less widespread. On the other hand, nowadays, continuous cover forestry is being reinvented, and in modern forestry it can be considered as a further development in sustainable forest management^9,10^. Hungary is a good example for this process, where despite its historical occurrence, this practice has only been recently re-implemented in few selected forestry districts^10^.

Ecosystem processes in forests are rooted in the functioning of the vegetation, which in turn affects animal communities, including arthropods, at different trophic levels in a cascading manner. However, changes in arthropod communities exert feedback and may have additional impact on ecosystem processes^11,12^. Spiders play an important role in forest ecosystems by their predatory action both on herbivorous insects and on other actors in the forest food web and thus contribute to the stability of forest ecosystems^12,13^. Moulder and Reichle^14^ in their classic study followed the fate of radioactive ^137^Cs isotopes through the food chain of a *Liriodendron* forest. They showed that spiders were the most important predators of the forest litter community both in numbers and in biomass. Spider predation may also result in cascading reactions in the forest food chain. For instance, predation on decomposers can alter litter decomposition and nutrient dynamics^15^ and may also play part in controlling herbivorous insects in the canopy^16^. In removal experiments, lack of spiders induced an increase in the populations of both herbivorous prey and smaller predatory arthropods^17^.

Spiders, being an integral part of forest ecosystems, are exposed and sensitive to both the abiotic and biotic changes in forests^18–21^. It is therefore important to investigate how spider communities respond to management practices that alter both the abiotic and the biotic components of the forest environment. Previous research indicated that homogeneous, even-aged plantations modify spider communities^22^, which change characteristically during the plantation growth cycle. In a semi-deciduous Atlantic forest region in Argentina^23^ spider richness rapidly increased from the point of pine plantation establishment. In the same plantation the initially very low proportion of native spider species increased from 6 to 37% during the first 6–7 years. When native forest types are managed by various forestry practices, then clear-cutting proved to be the most detrimental to the original forest spider fauna. Forest specialist spiders were largely replaced with open-habitat species in clear-cut areas in a Finnish study^24^. In the same study increasing retention of trees caused less abrupt changes in the spider fauna. Similarly, a Canadian study found that removal of 25–33% of trees shifted a characteristic old-growth spider fauna towards one, more typical of clear-cuts^25^. These studies indicate that drastic logging practices alter original forest-dwelling spider communities in general. To gain a more applicable understanding of the process, it is important to compare concrete management alternatives. In such an endeavour it should be established in replicated field experiments how management types, belonging to both rotation and continuous forestry systems, affect local and stand-wise spider diversity.

The Pilis Forestry Systems Experiment (https://piliskiserlet.ecolres.hu/en)^26,27^, mentioned as ‘Pilis Experiment’ for short, was implemented to compare the long-term effects of forestry interventions on forest site conditions, natural regeneration, and forest biodiversity. In the framework of this forest ecological experiment, in a randomized complete block design, we compared the prevalent silvicultural treatment types of the region. These included, apart from (1) untreated control areas, treatments of the regionally dominant even-aged rotation forestry management types, such as (2) clear-cutting, (3) clear-cutting with retention tree group and (4) the preparation cutting stage (with 30% removal) of the shelterwood system. As the most common uneven-aged management type we also had (5) gap-cutting, which is a practice in recently introduced continuous cover forestry^28^. The Pilis Experiment was established with a pre-treatment year, when full-scale observations and sampling took place. After the implementation of the forestry treatments the experiment continued using the same methodology to follow changes in microclimate, soil, vegetation and invertebrate fauna. Results concerning the first 1–3 post-treatment years have been published on soil, litter, microclimate^26,27^, understory vegetation^29^, tree regeneration^30^ and enchytraeid worms^31^. A comparative study on the combination of various plant and animal taxa, including spiders, was also published^32^. These initial results revealed that most of the ecological processes have been driven by species- and group-specific reaction to environmental changes caused by the treatments^26^. Tree removal lead to the establishment of ruderal and open habitat plant species in the clear-cut areas^29^. Retention tree groups could maintain legacies of the understory composition for some years^29^, but because of their drying effect they had a strong negative effect on components of the soil fauna^31^. For many groups, gap-cutting represented the best compromise between the objective of timber production and the negative environmental impact caused by the forestry treatment^29,32^.

In the present study, we give an account of how forestry treatments affected the ground-dwelling spider communities during the first five years of the Pilis Experiment. Our main goal was to establish both in absolute and in relative terms how the prevalent forestry interventions affect forest spider communities and what are the possible drivers of any change during the time-frame of the study. We report the trajectory of diversity and abundance changes in relation to the first pre-treatment year and compare different treatments during the post-treatment years. Both spider abundance and species richness showed a marginal increase in the treatment areas. Compositional shifts in the spider community were more pronounced than quantitative changes. In post-treatment years species composition in the treatment plots diverged from control plots, but compositions became similar again by the fifth year. These changes were correlated mostly to light intensity and humidity gradients, which resulted from the applied forestry treatments. Since treatments were implemented patchily in the experimental forest stand, it created heterogeneity that translated to a modest increase in both gamma and beta diversity. Beta diversity partitioning suggested that variance in local diversities resulted equally from richness difference and species replacement, which also suggests a quick species-level response to the altered environmental conditions.

## Results

Over the five years of the study we collected in total 7886 spider individuals, out of which 6246 were adults, identified to species level and 1640 juveniles, which were identified only to higher taxonomical levels and were excluded from analyses that required species level distinction. The adults belonged to 51 species from 17 families (Table S1).

**Figure 1:**
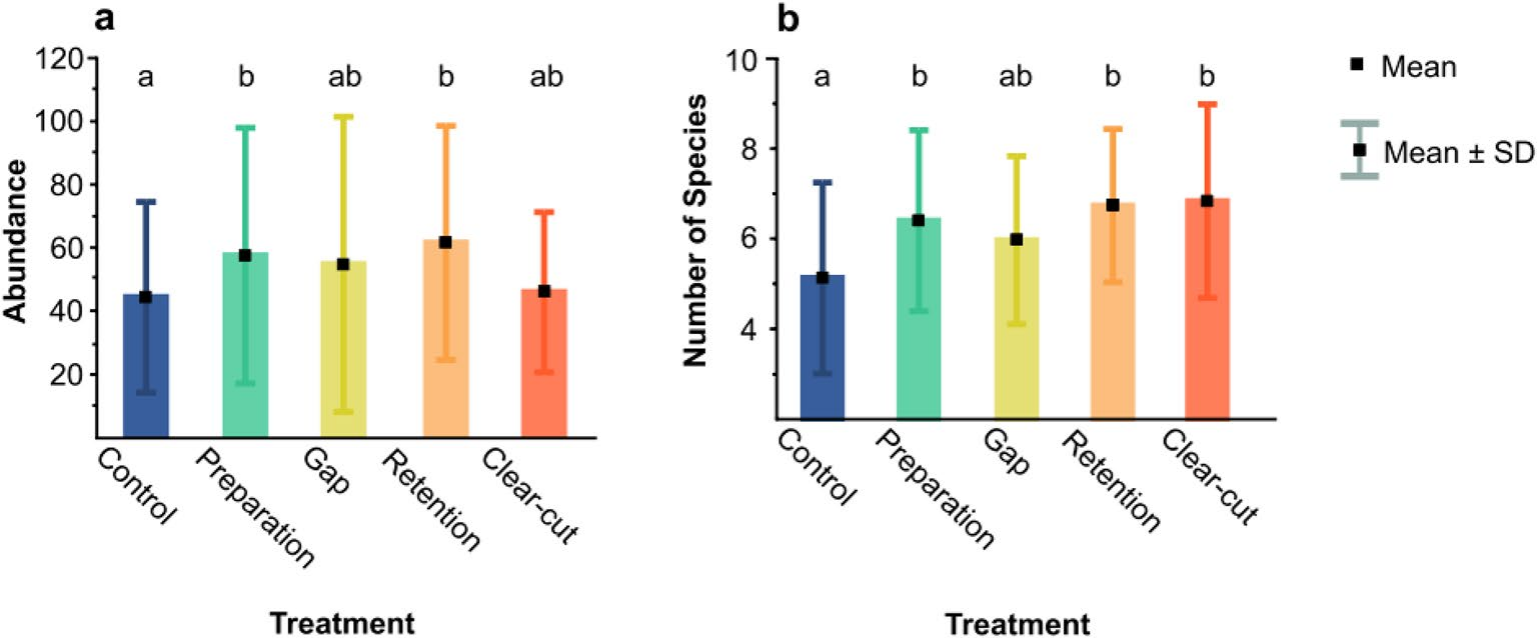
(**a**) Abundance and (**b**) species richness of spiders per plot per year in the different forestry treatments, considering only post-treatment years. Different letters signify significant difference at P < 0.05 in Tukey HSD test in the applied Linear Mixed-effect Models (see text for details).

**Figure 2:**
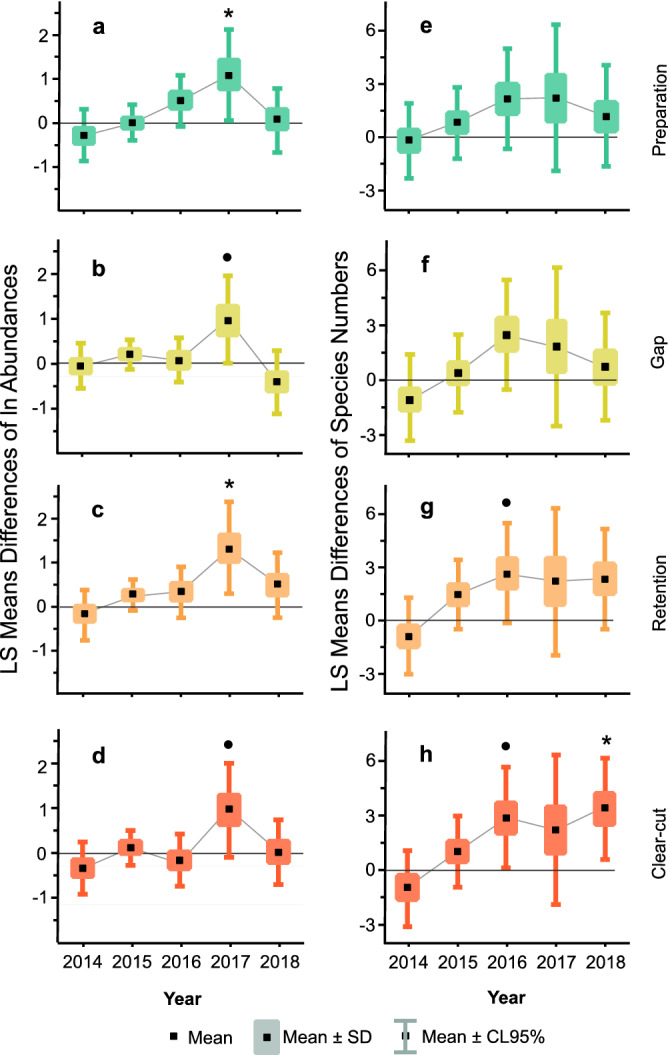
Least square mean differences between different treatments and control in ln(abundance) (**a-d**) and species richness (**e–h**) over the studied period. Significance of differences from control, separately for each year across the treatments, were evaluated by Tukey HSD test in the applied Linear Mixed-effect Models, with dots denoting marginal trends (0.05 < P < 0.1), asterisks denoting significance (P < 0.05).

Over the study years, spider abundance and species richness were affected by the forestry treatments. If we consider all post-implementation years (2015–2018), the treatment effect was significant for both spider abundance and species richness in the performed Linear Mixed-effect Models (LMMs, effect on ln(abundance): F = 5.09; d.f. = 4, 106.1; P = 0.0008; effect on species richness: F = 6.01; d.f. = 4, 106.3; P = 0.0002). The Tukey HSD test indicated that compared to the control, spider abundance was significantly higher in the preparation cutting and retention tree groups (Fig. 1a), whereas species richness was higher in all treatments except for gap-cutting (Fig. 1b). However, if comparisons to control were made separately for each year, these indicated that spider abundance and species richness in virtually all treatments rarely differed significantly from control (Fig. 2). Within years, we found virtually no difference between non-control treatments (Table S2), i.e. abundance and species richness was rather homogeneous across the different forestry management plots.

**Figure 3:**
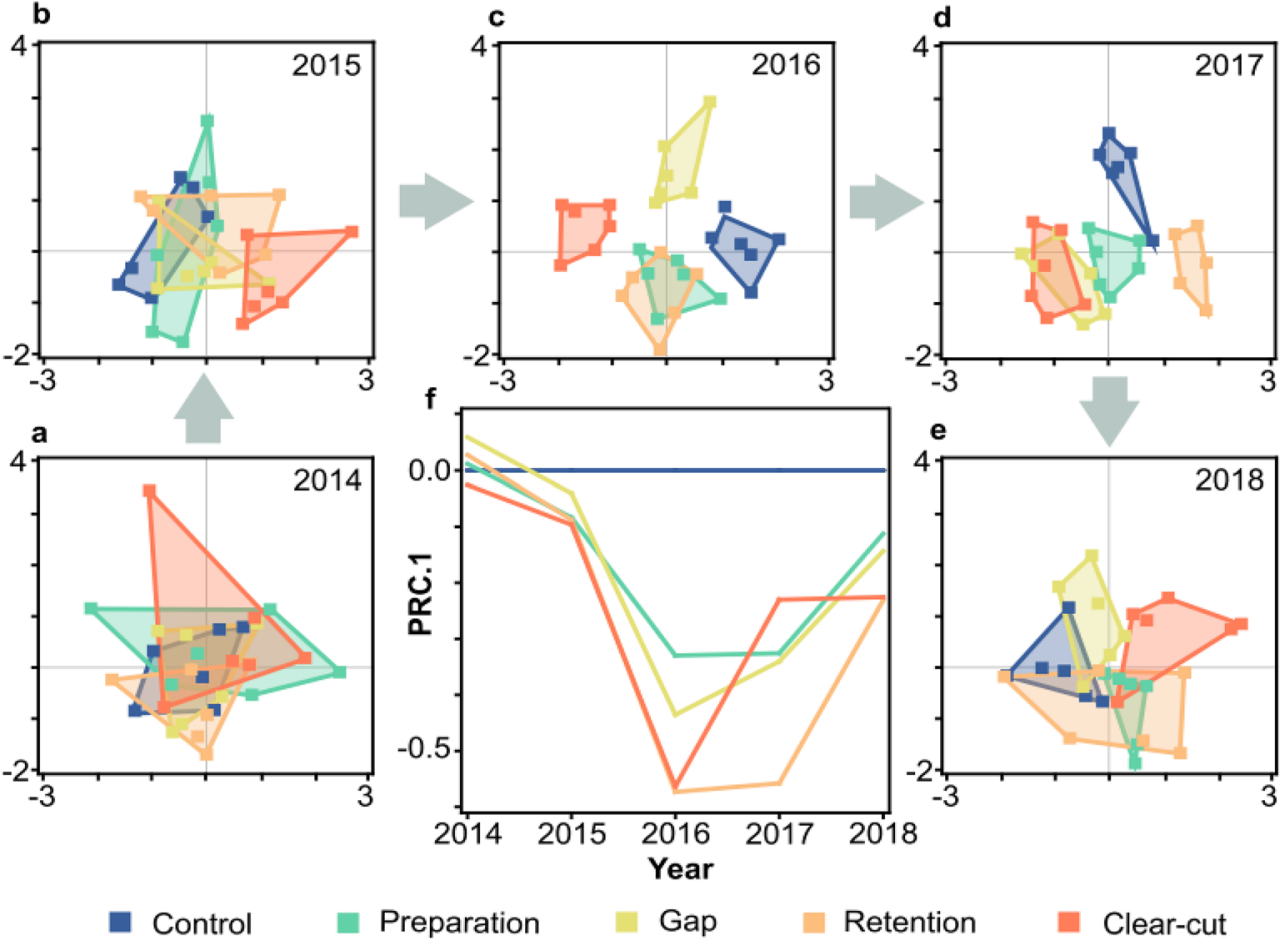
Unconstrained ordination plots of the spider communities in different treatment plots over the study years. (**a-e**) Principal Components Analysis ordination of treatment plots, grey arrows indicating the sequence of years; (**f**) Principal Response Curves plot of treatments relative to control. Treatments are colour coded according to the legend. The first two axes of the PCAs explained cumulative variation between 2014 and 2018 as follows: 41.71%, 48.53%, 68.19%, 56.63% and 44.10%, respectively. PRC.1 axis explained 64.01% of fitted variation.

Forestry treatments affected the species composition of spider communities to a considerable degree. Changes in community composition were examined by unconstrained linear ordination (Principal Components Analysis, PCA), executed yearly. The PCA for 2014 indicated that in the pre-treatment year spider communities of the prospective treatment plots largely overlapped (Fig. 3a). After the implementation of the treatments, from 2015 on, spider communities of plots in the different treatments started to diverge. This compositional dissimilarity reached its maximum in the 2nd-3rd year after treatments, after which, in the final observation year, communities became more similar (Fig. 3b-e). This course is captured well by a Principal Response Curves (PRC) analysis, which shows on one ordination axis how communities in different treatments over the years departed from communities in the control and became similar again (Fig. 3f). The different course of communities in treatments compared to control was significant as shown by the significance of the treatment × year interaction (Monte Carlo permutation test: F = 1.0, P = 0.001).

According to an Indicator Species Analysis with year as blocking factor, retention tree groups created the most specific conditions for spiders, having three indicator species (*Xysticus sabulosus, Harpactea rubicunda, Zodarion rubidium*) that preferentially occurred in these plots. *Drassyllus villicus* was a marginally significant species of the clear-cuts (Table 1). Of all four aforementioned indicator species, it can be said that they were either not present or only with few individuals in the pre-treatment control habitats, and showed a subsequent increase in their abundance over the years (for species specific responses see Figure S1). Another dominant species, *Pardosa lugubris*, also increased in its abundance over the years in treated plots, but not in the control ones, indicating a positive response to the changes caused by the forestry treatments. *Urocoras longispina* is an example for an opposite response to the treatments. This species was originally abundant in the pre-treatment plots, but its abundance decreased over post-treatment years in treated plots, while maintaining a relatively high abundance in control plots (Figure S1).

**Table 1:** Indicator Species Analysis results for the 10 most dominant species. Species with significant and marginally significant Indicator Values are highlighted in bold font. For family and author of species see Table S1.

Dominance rank	Species	Treatment	Indicator Value	Monte Carlo Mean	Monte Carlo S.D	P
1	*Urocoras longispina*	Control	23.7	22.6	1.56	0.2114
2	*Pardosa lugubris*	Gap	23.9	21.9	2.30	0.1783
3	*Trochosa terricola*	Preparation	22.5	22.6	1.70	0.4606
**4**	***Drassyllus villicus***	**Clear-cut**	**20.3**	**14.2**	**3.30**	**0.0532 ·**
5	*Histopona torpida*	Control	14.4	12.1	2.99	0.1902
**6**	***Xysticus sabulosus***	**Retention**	**42.5**	**20.1**	**3.32**	0.0001 ***
**7**	***Harpactea rubicunda***	**Retention**	**46.7**	**14.0**	**3.52**	0.0001 ***
8	*Agroeca brunnea*	Gap	9.9	10.4	2.74	0.4898
**9**	***Zodarion rubidium***	**Retention**	**18.3**	**10.5**	**3.74**	0.0414 *
10	*Dysdera erythrina*	Control	17.8	13.2	7.07	0.2126

**Figure 4:**
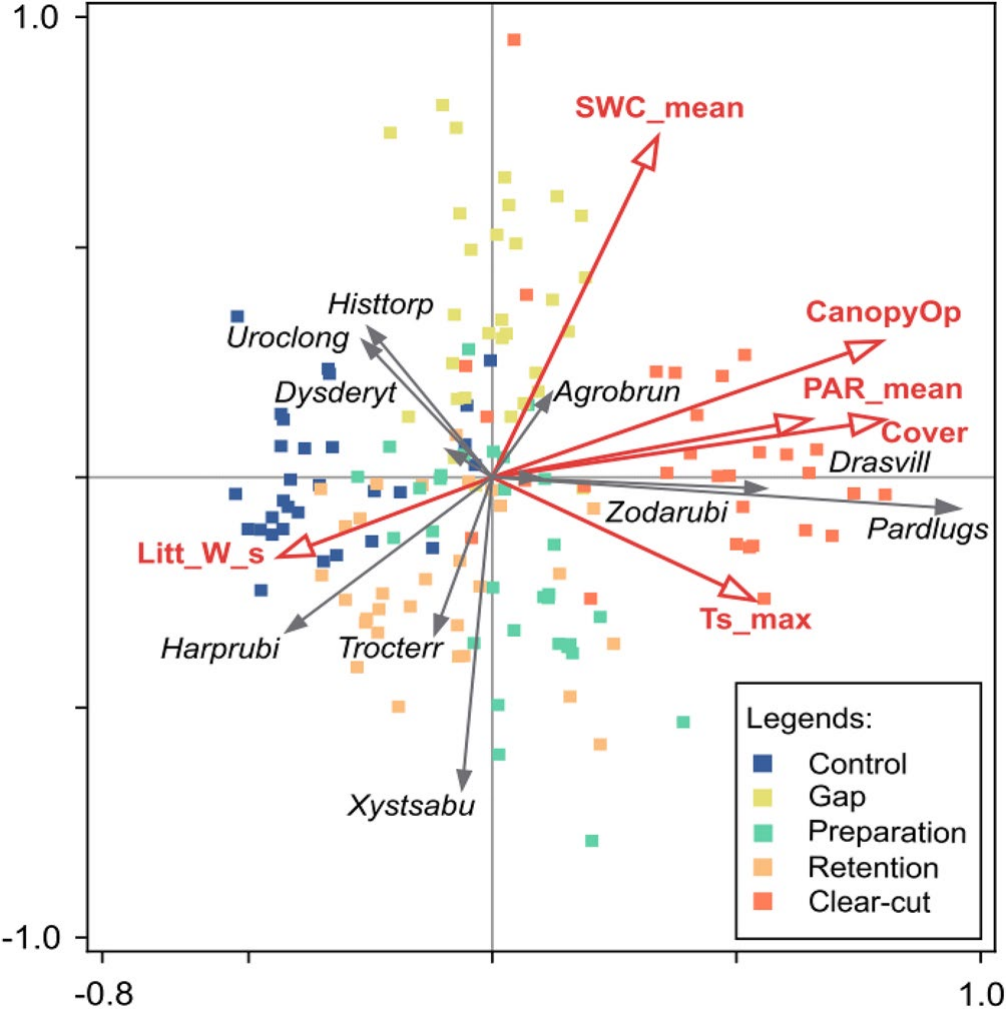
Ordination tri-plot of RDA analysis on the response of the 10 most dominant spider species to the environmental conditions of plots (separately for the experimental years), with block and year included as co-variables in the analysis. The yearly position of plots in the ordination space is colour coded according to forestry treatments. Abbreviated species (for family and author see Table S1): Uroclong—*Urocoras longispina*; Pardlugu—*Pardosa lugubris*; Trocterr—*Trochosa terricola* ; Drasvill—*Drassyllus villicus*; Histtorp—*Histopona torpida*; Xystsabu—*Xysticus sabulosus*; Harprubi—*Harpactea rubicunda*; Agrobrun—*Agroeca brunnea*; Zodarubi—*Zodarion rubidium*; Dysderyt—*Dysdera erythrina*. See Table 2 for resolving abbreviated environmental variable names.

We also investigated what kind of microclimate-, vegetation- and soil-related changes might have led to the specific responses of the 10 dominant spider species by the application of constrained ordination (Redundancy Analysis, RDA). These analyses were based on the data set including data from all five years and considering explanatory variables in Table 2 (mean and range of environmental variables per treatment, per year are given in SI Table 3). First, we ran a preliminary partial RDA with all explanatory variables, plus year and block as co-variables. This indicated that the simple effects of the variables ‘maximum air temperature’ and ‘maximum relative humidity’ were not significant at P = 0.05. Second, we ran the same analysis excluding these non-significant variables. We compared the explanatory power of this second RDA to the equivalent partial unconstrained analysis (PCA) with the same co-variables. In unconstrained ordination, the first two axes of PCA explained 44.76% of variance in species occurrence data. The RDA on the first two axes was responsible for 83.4% of the cumulative variation that was explained by the unconstrained analysis. Photosynthetically active radiation, soil moisture and the cover of understory vegetation had significant conditional effects. The statistics of all simple and conditional effects are given in Table S4. The constrained ordination revealed that the first major gradient, along which spider species responded to forestry management, was basically light or light-dependent variables, such as canopy openness, understory cover and soil temperature. Light represented one side of the gradient, as opposed to higher litter volume on the opposite side. The gradient of the second axis was related to soil moisture (positive direction) and in part to soil temperature (negative direction) (Fig. 4).

**Table 2:** Environmental variables used in constrained ordination analysis to explain species responses to environmental gradients, including microclimate and vegetation related parameters, measured at different strata in the experimental plots.

Variable	Unit	Measurement	Measurement height (cm)	Description
SWC_mean	m^3^/m^3^	Logger, daily mean	-20–0	Soil water content, daily mean
RH_max	%	Logger, daily max	130	Relative humidity, daily maximum
CanopyOp	%	Spherical densiometer	130	Canopy openness
PAR_mean	µmol·m^-1^·s^-1^	Logger, daily mean	150	Photosynthetically active radiation, daily mean
Ts_max	°C	Logger, daily max	-2	Soil temperature, daily maximum
T_max	°C	Logger, daily max	130	Air temperature, daily maximum
Litt_W_s	g/m^2^	Measuring sample	0	Litter dry weight in spring
Cover	%	Assessment in 2 m × 2 m quadrats	0–50	Understory cover

Clear-cutting and control treatments were separated along the first gradient (light–litter). The second axis separated gap from retention tree group and preparation cutting treatments following the moisture gradient. Among the dominant species *Pardosa lugubris* and *Drassyllus villicus* became more abundant in response to canopy openness and higher soil temperatures. With both species we could observe that their abundance was lower in the control treatment and variably higher in the forestry treatments, which all increased canopy openness to certain degrees (Figs. 4, S1). *Harpactea rubicunda* and *Trochosa terricola* responded with population decrease to the same gradient. These species were negatively associated with canopy openness and positively associated with litter volume and they had a constantly high presence in the control plots over the years. *Agroeca brunnea* showed a preference for more humid plots of the gaps, responding with higher abundance to the second gradient, while *Xysticus sabulosus* and also to some extent *T. terricola* showed an opposite response by being associated with dryer plots, with variable association with the retention tree and preparation cutting treatments. Two species, *Urocoras longispina* and *Histopona torpida*, known for building their webs under logs, stones and soil depressions, were associated with control plots and were negatively correlated with soil temperature and light. Over the years, both species showed an overall declining abundance (Fig. S1).

**Figure 5:**
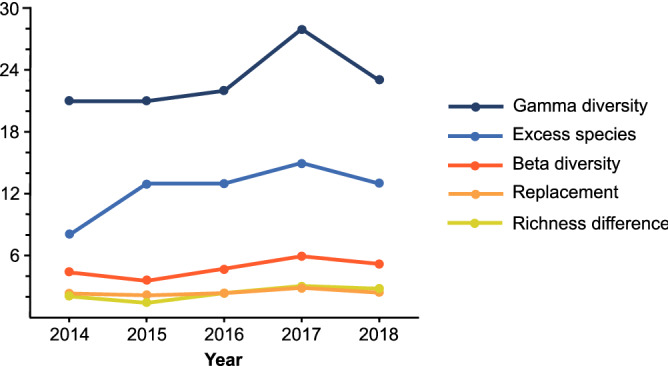
Changes of different diversity measures over the experimental years in the whole forest stand. 2014 was a pre-treatment year, 2015–2018 are experimental years after treatment implementation. Refer to Materials and methods for the description of diversity measures.

The different forestry treatments implemented in replicates within the investigated forest stand introduced spatial heterogeneity that left a mark on the spider community of the entire stand (Fig. 5). Total species number (gamma diversity) showed a moderate increase. We can define excess species richness as the contribution of non-control plots to total species richness. In the first, pre-implementation year this contribution arose from untreated plots, and was considerably smaller than in the later post-treatment years. Beta diversity, on the other hand, increased only slightly, with species replacement and richness difference contributing to a similar degree (Fig. 5).

## Discussion

As a result of the silvicultural treatments, both spider abundance and species numbers showed a marginal increasing tendency compared to the control plots and the pre-implementation state. However, forestry treatments affected spider communities mostly by altering their species composition. Species level changes underpinned these trajectories and these changes could be linked to species responses to altered environmental conditions, primarily that of the amount of light and humidity. Overall, forestry treatments applied in smaller blocks of the forest stand moderately increased gamma and beta diversity over the investigated period.

Forestry treatments result in a specific disturbance, which alters vegetation structure and microclimate. The interventions in the Pilis Experiment led to an increase in spider species richness in all treatments if we compare these to control (significant for all treatments except for gap-cutting). In treatments that resulted in a relatively small alteration to the original forest structure, specifically in retention tree groups and in preparation cutting, spider abundance was slightly higher than in control. However, in other treatments such a difference could not be shown. Intermediate disturbance hypothesis^33^ predicts that moderate changes in a habitat will increase diversity, because a moderate disturbance introduces new niches, creates more patchiness allowing for the coexistence of more species. Similarly, cyclic disturbance may create a balance between coloniser and competitive species, also contributing to higher species richness^34,35^. In a Canadian experiment^36^, spider species richness was higher in areas where various dispersed retention harvesting practices were applied, as compared to untreated control. This relationship held over all harvesting intensity treatments, in both deciduous and pine forests. However, more aggregated retention schemes had variable impact on spider species numbers^36^. Increased spider species richness was also observed when forests were subject to other physical disturbances, such as windthrows^18^. On the other hand, more severe disturbances that cause near complete habitat destruction, such as burning, had an unequivocal negative short- and medium-term effect on spider diversity^37^. Considering the range in scale and severity of the various forest disturbances in similar studies, the present set of treatments fits into the moderate disturbance category and may at least partially explain the observed increase in spider abundance and diversity.

It is important to consider the temporal scale of the disturbances and their effects. In the present experiment, we observed relatively little change in the first year and the greatest alterations were in the second and third post-treatment years. Since species responses, such as survival-extinction or immigration-emigration are not instantaneous, such a lag in community response can be expected, and have been reported in other studies^37,38^. The time frame when the effect of disturbances can be detected varies in different reports. For instance, ground-dwelling spiders’ species composition substantially diverged between ancient and recent forest sites in French oak-beech-dominated mature forests^39^, which is a scale of hundred years. Spider communities changed according to the growth cycle in pine plantations with 15 years forestry cycle^23^. A literature survey on clear-cutting found that in deciduous forest stands where harvesting techniques with a range of disturbance levels were applied, after two years, spiders responded with an increase in species richness, but with variable degrees in the different treatments. Species richness differences levelled out by the seventh year after the disturbance^40^.

The spatial scale and degree of patchiness of disturbances interact with their temporal effect. In boreal forests, clear-cutting homogenised forest environments across all spatial scales and ground-dwelling arthropod beta diversity reflected these changes at coarser scales^41^. At finer scales, spillover is an important process that alters species distributions, e.g. on the border of forests and open habitats^19^. Disturbance may also create habitat boundaries, and corresponding edge densities change with spatial scale. Edge effects also change local diversity by harbouring exclusively edge-associated, ecotone species, as was found in gaps created in oak forests^42^.

The trajectory of changes in the Pilis Experiment fits with the studies that observed a quick response from the spider community to forestry treatments. We argue, that this temporal pattern is very much a result of an interaction between spider traits, the spatial extent and the severity of treatments. Therefore, we predict that if treatments had caused greater habitat destruction and/or had they occurred on larger areas, we would have observed lower spider abundance and diversity in the given time frame. We also argue, that aggregate community measures (abundance and richness) differed imperceptibly between non-control treatments due to the species’ ecological traits. In particular, the high dispersal power of spiders and their functionally diverse regional species pool made possible a quick adaptation of local communities to the different treatments, resulting in a negligible change in abundance and richness values. Thus, at the given spatial extent and disturbance level the spider meta-community allowed a quick (but not complete) bouncing back of the local spider communities, thereby contributing to the maintenance of diversity and system stability. This argument is supported by findings that at the present scale of treatments we could observe differential responses in groups with contrasting ecological traits. For instance, enchytraeids with lower dispersal ability and higher sensitivity to environmental changes responded much more differentially to the treatments^32^.

Behind the moderate changes in species richness there was a considerable transformation of species compositions in the different treatments. Community compositions that completely overlapped in the pre-treatment year, deviated from control to the greatest extent in the second-third year, but became more similar by the last year of the observations. Other studies also indicate that differences in habitat structure are readily translated to differences in the species composition of spiders without much quantitative difference in abundance or species richness^22^. While it can be said that treatments induced definite compositional changes in the spider communities, we could not pinpoint any treatment that would have had a stronger effect than the others. Not even clear-cutting, regarded to be the most severe, distanced species composition considerably more than the other treatments, as was revealed by the overlapping trajectories in PRC analysis. In this regard, gap-cutting, the least severe continuous cover forestry intervention, also caused a similar deviation from control spider communities, than the other, more drastic treatments.

We argue that forestry interventions seem to affect spider communities more indirectly by altering vegetation structure, microclimate and other forest site characteristics. Monitoring vegetation and microclimate changes revealed that species association with plots was correlated with two major environmental gradients that came along with the forestry treatments. The amount of light – positively correlated with canopy openness and negatively with litter volume – proved to be the most important environmental gradient. Increased light was the most prominent in the clear-cut areas, and, as expected, remained at original level in the control plots. The higher amount of light resulted in a species rich and dense understory vegetation in the clear-cut plots^29^. Moisture came as the second most important factor. Retention tree groups, remaining in the clear-cut areas, maintained a strong evapotranspiration, and dried the soil to a considerable extent. The opposite effect, lower evaporation and transpiration rates in the partially shaded areas of gaps, resulted in moister soil conditions in those plots^26^.

In the present study, and also in another study^43^, spider species gave specific responses to the environmental gradients created by the forestry treatments. In the Pilis Experiment, the interventions in all treatments induced changes along the main gradients of light level and humidity. Responding to these gradients, there were species that were originally abundant in the pre-treatment plots, but declined over the consecutive years in the treatment plots, while maintaining a relatively high abundance in the control plots. There were other species that showed an opposite reaction and over time increased their abundance in treatment plots. Environmental conditions may act as a filter, and result in specific distribution of species in certain habitat types. For instance, in Germany *Drassylus villicus* or *Pardosa lugubris* occurs preferentially in more open, oak-dominated forest plots^44^. Here they occurred preferentially in the clear-cut areas offering similar, open conditions. In a Hungarian study *Urocoras longispinus* was associated with shaded, humid stands^45^; here it was typical for the control areas. Altered environmental conditions may also influence the distribution of spiders indirectly, for instance, through inducing possible changes in prey availability^46^. This might explain the occurrence of *Harpactea rubicunda* in control plots with higher litter volume, where woodlice, its preferential prey, is more likely to occur.

Considering the entire forest stand, the heterogeneity created by the patchy interventions slightly increased both gamma diversity and beta diversity. Forest management may occur at several scales. Recently finer-grain interventions are becoming preferred, because these may better preserve biodiversity^7^. While large-scale clear-cutting was shown to homogenise the forest environment^41^, smaller scale management implementations can be expected to increase habitat heterogeneity and patchiness. We propose that the observed changes in both gamma and beta diversity are due to increased patchiness within the forest stand. The created heterogeneity at local scale is likely to be reflected in the richness difference component of beta diversity, whereas we observed that species replacement played a role of similar importance. In studies with wider regional cover, and therefore wider range of habitat differences, species replacement was more prominent in forest sites^47^. Spider meta-community analysis have shown a largely coherent distribution of spiders^48^. In the case of a coherent distribution, species in the meta-community respond promptly to locally manifesting environmental variation through the process of habitat filtering^47^.

The high dispersal capacity of spiders allows their occupancy of suitable habitat patches^48^. Dispersal and habitat filtering contribute to species replacement. However, in the case of finer-grain habitat patchiness, edge effect and spillover are processes that counteract fixed species responses to environmental conditions^49^. We suggest, that in the present study such fine-grain processes are the reason why richness difference and replacement components of beta diversity had the same weight. Being able to coherently trace environmental changes, and being a ubiquitous arthropod group in all forests, spiders are rendered to be a suitable indicator group of changes in forest ecosystems. However, the sensitivity and distinctive power of spider indication seems to depend on the scale of the disturbances. The quick recovery of spiders after relatively small-scale disturbances added to the stability of the ecosystem. Our study also exemplified the importance of considering which spatial scales are optimal for silvicultural treatments that simultaneously achieve forestry objectives and only minimally affect ecosystem integrity.

## Materials and methods

### Study area

The Pilis Forestry Systems Experiment was located in a 40 ha sized, 80 year old oak–hornbeam forest stand (centre point: 47°40′28"N, 18°54′28"E) in the Pilis Mountains in the vicinity of Pilisszántó, Hungary. The elevation of the area is 370–470 m a.s.l., average annual mean temperature is 9.0–9.5 °C, with mean annual precipitation of 600–650 mm. The bedrock consists of limestone and red sandstone with loess. The dominant soil type of the area is luvisol and rendzic leptosol^27^. The stand is a primary forest, but it has been regularly managed, recently by shelterwood silvicultural system resulting in an even-aged, structurally homogenous stand. The canopy layer was dominated by sessile oak (*Quercus petraea* (Matt.) Liebl.), with a mean tree height of 21 m and mean diameter at breast height of 28 cm. The canopy layer also contained as subdominant species turkey oak (*Quercus cerris* L.), beech (*Fagus sylvatica* L.) and wild cherry (*Prunus avium* L.). Hornbeam (*Carpinus betulus* L.) formed a secondary canopy layer at 11 m height, where manna ash (*Fraxinus ornus* L.) appeared as a subordinate species. The shrub layer was scarce with a herbaceous understory cover of c. 30%, dominated by *Carex pilosa* Scop. and *Melica unflora* L.

### Treatments and experimental design

**Figure 6:**
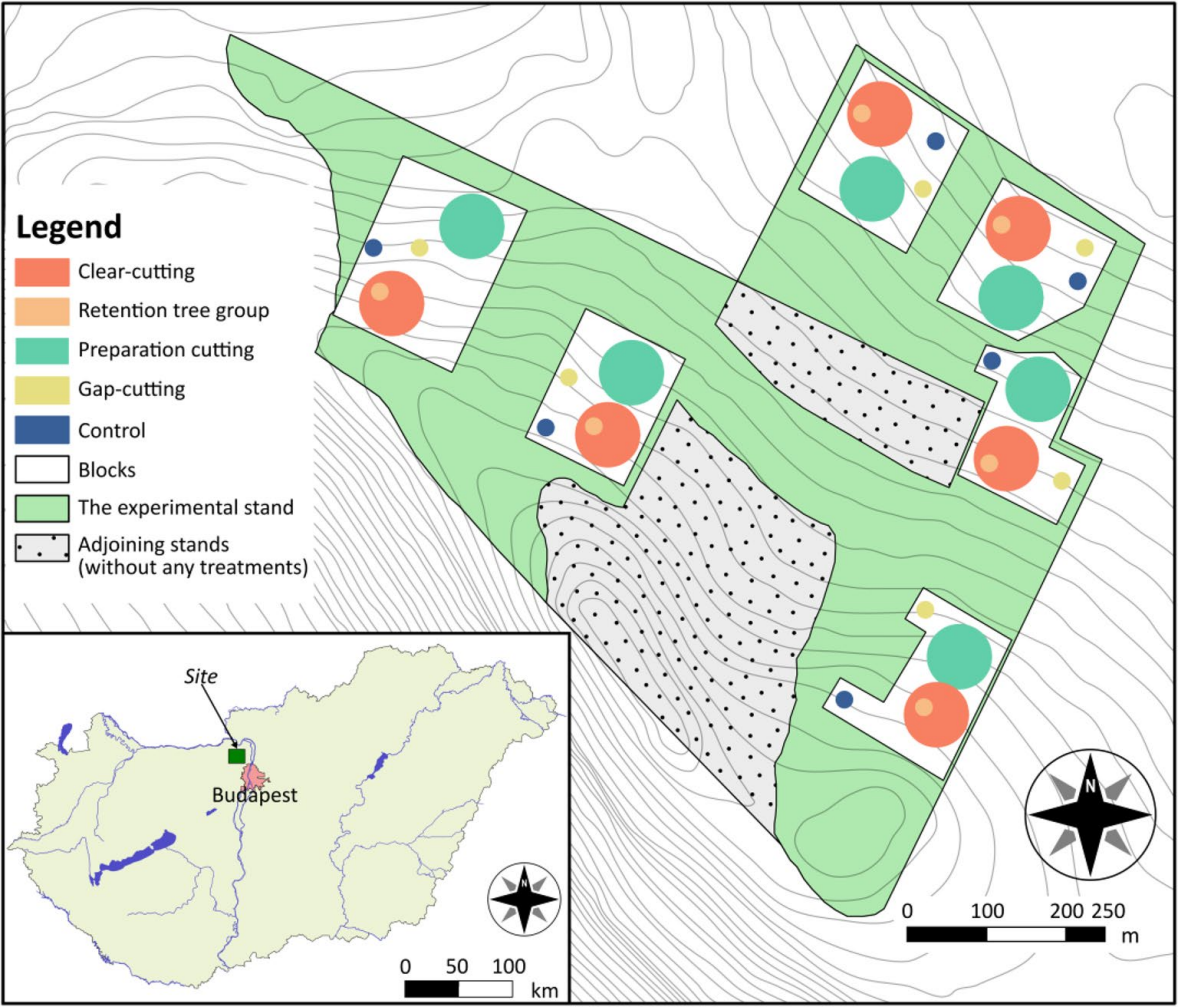
Overview of the experimental setup depicting the whole stand, the six replicate blocks and the treatment areas within the blocks. Treatments are colour coded according to legend.

We investigated the effects of four forestry interventions, compared to uncut control parts of the stand, constituting five treatments in the statistical sense. These were as follows: control, clear-cutting, retention tree group, uniform preparation cutting and gap-cutting. Preparation cutting and clear-cutting, often with retention tree group, represent characteristic stages of the even-aged, rotation forestry system, while gap-cutting is a frequently used management type in continuous cover forestry. The four treatments were implemented between December 2014 and January 2015 in the forest stand in six replicates (blocks) (Fig. 6). Details of the implemented treatments were the following: 1. Control: closed canopy stand without any treatment; 2. Clear-cutting: a 0.5 ha circular clear-cutting area of 80 m diameter, surrounded by closed canopy stand; 3. Gap-cutting: an artificial circular gap in the closed stand (20 m diameter, approx. one height/diameter ratio); 4. Preparation cutting: 30% of the total basal area of the dominant tree layer and the whole secondary tree layer was removed in a spatially uniform way in a circle of 80 m diameter; 5. Retention tree group: a circular group of upper canopy trees (20 m diameter, 8–12 dominant individuals, untouched subcanopy layer) in the clear-cutting. Treatment plots comprised the centre of the implementation areas. In the plots a 6 × 6 m area was fenced to prevent large herbivore grazing, which was necessary for certain botanical observations. Arthropod sampling in each plot was performed outside the fenced areas, c. 1 m from the fence.

The implementation of the experiment followed a randomized complete block design (Fig. 6). In this design plots of each of the five treatments (four treatments plus control) occurred once in each of the six blocks with random placement. This amounted to a total number of 30 plots. The area of the plots was already designated in 2014. This way, still in the uncut stand, we recorded the before-treatment state of each plot. With interventions carried out in the winter of 2014–2015, from 2015 on the plots received no further treatment, natural regeneration and regrowth processes were allowed to take their natural course.

### Data collection and processing

Data collection followed the concept of Before-After Control-Impact experiments^50^, recording all investigated variables and executing arthropod sampling in the vegetation period of the pre-treatment year (2014) and repeating the measurements and samplings in every post-treatment year (2015–2018).

During the whole study period, we recorded environmental variables related to microclimate, soil and the vegetation. Environmental variables considered in the present study are summarised in Table 2. The impact of forest management on the microclimate together with detailed methodological description of data collection has been published by Kovács et al.^26^. Here only a brief account of the measurements is given. Microclimate variables were systematically measured in the centre of each plot using four-channel Onset HOBO H021-002 data loggers (Onset Computer Corporation, Bourne, Massachusetts, USA) in each year during the growing season (March–October). In each month, a 48 h period was measured with 10 min logging intervals. Using the specific sensors of this equipment, we measured photosynthetically active radiation (PAR), air temperature (T), relative humidity (RH), soil temperature (Ts) and soil water content (SWC) (Table 2.).

The collected and manually screened 48 h long stream of microclimate data was split into two 24 h subsets, out of which one was randomly chosen for further analysis. The chosen 24 h subset was the basis for the calculation of daily mean and maximum values representing the given month. These daily mean or maximum values of the respective variables from each month were averaged to result a yearly figure to describe the microclimatic conditions of the plot (see SI Table 3 for complete list of values). For the variables we used either the mean or the maximum values based on their utility to separate the different treatments, relying on the results of Kovács et al.^26^.

The surveys of vascular plants were carried out in a 2 × 2 m sized quadrat in each plot placed right outside the fenced 6 × 6 m area, close to the pitfall traps. The cover of understory plant species was estimated in percentage, arboreal individuals were included to the sampling only under 0.5 m height. The surveys were carried out yearly in spring (April) and summer (June). Data of the two aspects were merged using the maximum cover values of each recorded species. Canopy openness was measured by spherical densiometer in each year in the middle of the vegetation season^51^. Additionally, twice in each year (in spring and in autumn), the dry mass of litter was also measured, from which we used the spring data.

To sample ground-dwelling predatory arthropods (spiders (Aranae) and ground beetles (Coleoptera: Carabidae)), four pitfall traps were installed in every plot around the fenced area in each direction. In every year, there were two one month sampling intervals (in spring and in autumn), corresponding to the highest activity regime of the target groups^52^. The traps were made of 85 mm diameter plastic cups; each containing approximately 250 cm^3^ of a 50% solution of propylene glycol and water, saturated with salt and with a drop of odourless detergent to reduce surface tension. A dark green plastic roof protected the solution from litter and rain. Yearly data of the pitfall traps of the same plots were merged resulting 30 sampling units for each study year.

### Ethical approval

We conducted sampling from the spider and carabid assemblages with maximal respect to animal welfare. All applicable international, national, and/or institutional guidelines for the care and use of animals were followed. The observation of plant communities in this study were non-invasive, while the field sampling of invertebrates such as spiders and ground-beetles were conducted under the license from the respective Hungarian authority (Közép-Duna-Völgyi Környezetvédelmi és Természetvédelmi Felügyelőség KTF:30362-3/2014).

### Statistical analysis

For the univariate analyses we used the software JMP^53^. Ordination methods were applied using Canoco 5^54,55^, whereas Indicator Species Analysis was performed with PCOrd^56^.

Overall treatment effect on spider abundance (ln transformed) and species richness for the post-implementation years was analysed with Linear Mixed-effect Models (LMM), where, as random factors, we included year and block to account for spatial and temporal blocking effects. To assess the treatment effect attributable to forestry treatments in each year, LMMs including block as random factor were applied separately for each year, including the pre-implementation year. Post-hoc differences in treatment effects were assessed with the visualisation of Least Squares means differences to control, significance calculations of these multiple comparisons were based on Tukey HSD test.

The response of spider species composition to the unfolding effect of the silvicultural treatments over the observation years was examined by unconstrained ordination analyses^54^, including only the 10 overall most dominant spider species. Canoco performs a Detrended Correspondence Analysis on the data, which analysis indicated a 2.5 SD unit long gradient in the species composition response data, according to which the usage of linear unconstrained ordination analyses, Principal Components Analysis (PCA) was used. To explore if we need to take into account block effect, a constrained partial Redundancy Analysis (RDA) with block as the only constraining variable was run. This revealed a modest spatial blocking effect, being responsible for 7.53% of the cumulative variation over the 4 axes included in the analysis. For this reason, Block was included as co-variable in all subsequent ordination analyses. Therefore, to depict community composition changes over time, we performed PCA analyses separately for each investigated year. To summarise yearly changes of the spider community in the various treatments relative to control, Principal Response Curves (PRC) analysis was applied^57^. PRC calculates scores for a single ordination axis based on the scores of an RDA, in which time (years in our case) was the covariate and the single explanatory variable was the interaction between treatment and time. Significance of the explanatory variable was tested with Monte Carlo permutation test.

To explore how the spider community responded to environmental gradients created in part by the silvicultural treatments, we performed constrained ordination, also in Canoco 5. Similarly to the unconstrained methods, a linear method, RDA was chosen. In these RDAs, we used data from all years as a single dataset, and included both block and year as co-variables. We started off with the environmental variables listed in Table 2. To judge the importance of variables, the analysis calculated simple (marginal) effects for each variable, which explained variance resulting from fitting only the single variable in question. Conditional effect of the variables was also reported, which is the explanatory contribution of a variable during a forward selection, when it is entered after other variables with greater explanatory power are already in the model. Significance of entered variables was tested by Monte Carlo permutation-based pseudo-F test. In calculating conditional effects, because of the large number of permutation tests performed, Canoco also reports adjusted Type I error estimates, P(adj), based on the approach of false discovery rates^54^. To screen out less important environmental variables, we first run a preliminary RDA with all variables included, and left out those variables from subsequent analyses which had non-significant marginal effect.

Beta diversity calculation between all treatment plots of the forest stand, separately for each year, was quantified based on the pairwise index of Weiher and Boylen^58^. Although this diversity index is not a frequently used one (see the review of Koleff et al.^59^), the essence of the index is that it expresses beta diversity in number of species, thereby allowing its direct comparison with other richness measures, as in Fig. 5. Following Podani and Schmera^60^, the Weiher and Boylen beta diversity was partitioned into replacement and richness difference components. We also calculated “excess species richness” separately for each year. Excess species richness was the number of species that were found due to the presence of treatment (non-control) plots. It was calculated by subtracting species richness of the control plots from total species richness (gamma diversity) of the whole forest stand.

## Supplementary Information

Supplementary Information.

## Data Availability

All data generated or analysed during this study are included in this published article (and its supplementary information files).
