# Supplementary material for: Resilience of spider communities affected by a range of silvicultural treatments in a temperate deciduous forest stand
Source: Sci Rep. 2021 Oct 15;11:20520. doi: 10.1038/s41598-021-99884-8 (PMC8520002; doi:10.1038/s41598-021-99884-8)
Supplement: Supplementary file 1 — Supplementary Information. [file 41598_2021_99884_MOESM1_ESM.docx]

**Supplementary Information to:**

**Resilience of spider communities affected by a range of silvicultural treatments in a temperate deciduous forest stand**

Ferenc Samu^1*^, Zoltán Elek^2^, Bence Kovács^3^, Dávid Fülöp^1^, Erika Botos^1^, Dénes Schmera^4^, Réka Aszalós^3^, András Bidló^5^, Csaba Németh^3^, Vivien Sass^5^, Flóra Tinya^3^, Péter Ódor^3^

(1) Centre for Agricultural Research, Plant Protection Institute, Eötvös Loránd Research Network, Herman Ottó út 15, Budapest, H-1022 Hungary;

(2) MTA-ELTE-MTM Ecology Research Group, Pázmány P. stny. 1/C, Budapest, H-1117 Hungary;

(3) Centre for Ecological Research, Institute of Ecology and Botany, Eötvös Loránd Research Network, Alkotmány u. 2-4., Vácrátót, H-2163 Hungary;

(4) Balaton Limnological Research Institute, Eötvös Loránd Research Network, Klebelsberg Kuno u. 3, Tihany, H-8237 Hungary;

(5) University of Sopron, Faculty of Forestry, Institute of Environmental and Earth Sciences, Bajcsy-Zsilinszky u. 4., Sopron, H-9400 Hungary.

**Table S1.** The list of spider species (with family affiliation) collected over the observation years in the Pilis Experiment in decreasing dominance order. Yearly and total number of individuals caught. Juveniles without species level identification are not indicated.

| **Family** | **Species** | **2014** | **2015** | **2016** | **2017** | **2018** | **Total** |
| --- | --- | --- | --- | --- | --- | --- | --- |
| Amaurobiidae | *Urocoras longispina* (Kulczynski, 1897) | 1185 | 295 | 518 | 175 | 126 | 2299 |
| Lycosidae | *Pardosa lugubris* s.str. (Walckenaer, 1802) | 49 | 112 | 630 | 896 | 46 | 1733 |
| Lycosidae | *Trochosa terricola* Thorell, 1856 | 161 | 141 | 243 | 80 | 189 | 814 |
| Gnaphosidae | *Drassyllus villicus* (Thorell, 1875) | 4 | 6 | 56 | 246 | 14 | 326 |
| Agelenidae | *Histopona torpida* (C. L. Koch, 1834) | 222 | 39 | 10 | 19 | 4 | 294 |
| Thomisidae | *Xysticus sabulosus* (Hahn, 1832) |  | 46 | 90 | 74 | 40 | 250 |
| Dysderidae | *Harpactea rubicunda* (C. L. Koch, 1838) |  | 14 | 28 | 39 | 9 | 90 |
| Liocranidae | *Agroeca brunnea* (Blackwall, 1833) | 31 | 3 | 10 | 8 | 14 | 66 |
| Zodariidae | *Zodarion rubidum* Simon, 1914 | 1 | 1 | 19 | 13 | 22 | 56 |
| Dysderidae | *Dysdera erythrina* (Walckenaer, 1802) | 44 | 4 | 1 |  |  | 49 |
| Thomisidae | *Xysticus kochi* Thorell, 1872 | 36 | 6 |  |  |  | 42 |
| Phrulolithidae | *Phrurolithus festivus* (C. L. Koch, 1835) |  | 1 | 10 | 21 | 2 | 34 |
| Atypidae | *Atypus affinis* Eichwald, 1830 | 7 | 7 | 7 | 2 | 4 | 27 |
| Lycosidae | *Alopecosa pulverulenta* (Clerck, 1757) |  |  | 8 | 6 | 2 | 16 |
| Zoridae | *Zora spinimana* (Sundevall, 1833) |  | 1 |  | 12 | 1 | 14 |
| Lycosidae | *Aulonia albimana* (Walckenaer, 1805) |  |  | 4 | 7 | 2 | 13 |
| Lycosidae | *Trochosa robusta* (Simon, 1876) | 13 |  |  |  |  | 13 |
| Gnaphosidae | *Drassyllus praeficus* (L. Koch, 1866) |  |  | 4 | 5 | 2 | 11 |
| Gnaphosidae | *Haplodrassus silvestris* (Blackwall, 1833) | 4 |  |  | 3 | 3 | 10 |
| Liocranidae | *Scotina celans* (Blackwall, 1841) |  |  |  | 3 | 6 | 9 |
| Gnaphosidae | *Zelotes erebeus* (Thorell, 1870) |  |  |  |  | 8 | 8 |
| Gnaphosidae | *Zelotes aurantiacus* Miller, 1967 |  |  | 6 | 1 |  | 7 |
| Clubionidae | *Clubiona terrestris* Westring, 1851 | 5 | 1 |  |  |  | 6 |
| Hahniidae | *Cicurina cicur* (Fabricius, 1793) | 2 |  | 2 |  | 2 | 6 |
| Dysderidae | *Dysdera ninnii* Canestrini, 1868 | 6 |  |  |  |  | 6 |
| Linyphiidae | *Diplostyla concolor* (Wider, 1834) |  |  | 3 | 3 |  | 6 |
| Liocranidae | *Apostenus fuscus* Westring, 1851 |  |  |  | 4 |  | 4 |
| Lycosidae | *Hogna radiata* (Latreille, 1819) |  | 4 |  |  |  | 4 |
| Pisauridae | *Pisaura mirabilis* (Clerck, 1757) | 1 | 1 |  | 2 |  | 4 |
| Lycosidae | *Pardosa luctinosa* Simon, 1876 | 3 |  |  |  |  | 3 |
| Gnaphosidae | *Drassodes lapidosus* (Walckenaer, 1802) |  | 1 |  | 1 |  | 2 |
| Gnaphosidae | *Drassodes pubescens* (Thorell, 1856) |  |  |  |  | 2 | 2 |
| Gnaphosidae | *Gnaphosa bicolor* (Hahn, 1833) | 1 |  |  | 1 |  | 2 |
| Gnaphosidae | *Trachyzelotes pedestris* (C. L. Koch, 1837) |  | 2 |  |  |  | 2 |
| Linyphiidae | *Trichoncus affinis* Kulczynski, 1894 |  |  | 2 |  |  | 2 |
| Agelenidae | *Allagelena gracilens* (C. L. Koch, 1841) |  | 1 |  |  |  | 1 |
| Agelenidae | *Tegenaria campestris* (C. L. Koch, 1834) | 1 |  |  |  |  | 1 |
| Clubionidae | *Clubiona neglecta* O. P.-Cambridge, 1862 |  |  |  |  | 1 | 1 |
| Linyphiidae | *Erigone dentipalpis* (Wider, 1834) | 1 |  |  |  |  | 1 |
| Linyphiidae | *Neriene clathrata* (Sundevall, 1830) | 1 |  |  |  |  | 1 |
| Linyphiidae | *Panamomops mengei* Simon, 1926 |  |  | 1 |  |  | 1 |
| Linyphiidae | *Porrhomma microphthalmum* (O. P.-Cambridge, 1871) | |  |  | 1 |  | 1 |
| Linyphiidae | *Tapinopa longidens* (Wider, 1834) |  |  |  |  | 1 | 1 |
| Linyphiidae | *Tenuiphantes flavipes* (Blackwall, 1854) |  |  | 1 |  |  | 1 |
| Linyphiidae | *Trichoncus hackmani* Millidge, 1956 |  |  |  | 1 |  | 1 |
| Linyphiidae | *Walckenaeria furcillata* (Menge, 1869) |  |  |  | 1 |  | 1 |
| Lycosidae | *Pardosa agrestis* (Westring, 1861) |  | 1 |  |  |  | 1 |
| Salticidae | *Evarcha arcuata* (Clerck, 1757) |  |  |  |  | 1 | 1 |
| Tetragnathidae | *Pachygnatha degeeri* Sundevall, 1830 |  |  | 1 |  |  | 1 |
| Theridiidae | *Asagena phalerata* (Panzer, 1801) |  |  |  | 1 |  | 1 |
| Thomisidae | *Ozyptila claveata* (Walckenaer, 1837) |  |  |  | 1 |  | 1 |

**Figure S1.** Yearly abundance changes of the most dominant spider species in the studied forest plots. Sub-figures of species are in dominance order.

**Table S2.** Least square means differences (Difference) of different treatments from control in ln(abundance) and species richness over the studied period. Significance of differences from control, separately for each year across the treatments, were evaluated by Tukey HSD test in the applied Linear Mixed-effect Models. Values of P < 0.05 are set in bold. Treatment levels: C = control, CC = clear-cutting, G = gap-cutting, R = retention tree group, P = preparation cutting.

| **Response** | **Year** | **Level1** | **Level2** | **Difference** | **Std Err Dif** | **Lower CL** | **Upper CL** | **p-Value** |
| --- | --- | --- | --- | --- | --- | --- | --- | --- |
| LnAbund | 2014 | CC | C | -0.33 | 0.202 | -0.929 | 0.279 | 0.5088 |
| LnAbund | 2014 | G | C | -0.11 | 0.202 | -0.713 | 0.496 | 0.9824 |
| LnAbund | 2014 | G | CC | 0.22 | 0.202 | -0.388 | 0.821 | 0.8181 |
| LnAbund | 2014 | G | P | 0.17 | 0.202 | -0.436 | 0.773 | 0.9170 |
| LnAbund | 2014 | G | R | 0.08 | 0.202 | -0.524 | 0.684 | 0.9944 |
| LnAbund | 2014 | P | C | -0.28 | 0.202 | -0.881 | 0.328 | 0.6527 |
| LnAbund | 2014 | P | CC | 0.05 | 0.202 | -0.556 | 0.653 | 0.9992 |
| LnAbund | 2014 | R | C | -0.19 | 0.202 | -0.793 | 0.416 | 0.8809 |
| LnAbund | 2014 | R | CC | 0.14 | 0.202 | -0.468 | 0.741 | 0.9592 |
| LnAbund | 2014 | R | P | 0.09 | 0.202 | -0.516 | 0.693 | 0.9918 |
| LnAbund | 2015 | CC | C | 0.12 | 0.140 | -0.295 | 0.542 | 0.9000 |
| LnAbund | 2015 | CC | P | 0.12 | 0.140 | -0.302 | 0.535 | 0.9165 |
| LnAbund | 2015 | G | C | 0.20 | 0.140 | -0.217 | 0.620 | 0.6086 |
| LnAbund | 2015 | G | CC | 0.08 | 0.140 | -0.340 | 0.497 | 0.9793 |
| LnAbund | 2015 | G | P | 0.20 | 0.140 | -0.223 | 0.613 | 0.6374 |
| LnAbund | 2015 | P | C | 0.01 | 0.140 | -0.412 | 0.425 | 1.0000 |
| LnAbund | 2015 | R | C | 0.27 | 0.140 | -0.152 | 0.685 | 0.3451 |
| LnAbund | 2015 | R | CC | 0.14 | 0.140 | -0.275 | 0.562 | 0.8406 |
| LnAbund | 2015 | R | G | 0.07 | 0.140 | -0.353 | 0.483 | 0.9896 |
| LnAbund | 2015 | R | P | 0.26 | 0.140 | -0.158 | 0.678 | 0.3691 |
| LnAbund | 2016 | CC | C | -0.15 | 0.202 | -0.754 | 0.454 | 0.9437 |
| LnAbund | 2016 | G | C | 0.06 | 0.202 | -0.548 | 0.661 | 0.9985 |
| LnAbund | 2016 | G | CC | 0.21 | 0.202 | -0.398 | 0.811 | 0.8418 |
| LnAbund | 2016 | P | C | 0.52 | 0.202 | -0.083 | 1.126 | 0.1119 |
| LnAbund | 2016 | P | CC | 0.67 | 0.202 | 0.067 | 1.276 | **0.0249** |
| LnAbund | 2016 | P | G | 0.47 | 0.202 | -0.139 | 1.069 | 0.1853 |
| LnAbund | 2016 | P | R | 0.20 | 0.202 | -0.404 | 0.804 | 0.8567 |
| LnAbund | 2016 | R | C | 0.32 | 0.202 | -0.283 | 0.926 | 0.5188 |
| LnAbund | 2016 | R | CC | 0.47 | 0.202 | -0.133 | 1.076 | 0.1751 |
| LnAbund | 2016 | R | G | 0.27 | 0.202 | -0.339 | 0.869 | 0.6873 |
| LnAbund | 2017 | CC | C | 0.97 | 0.362 | -0.117 | 2.051 | 0.0947 |
| LnAbund | 2017 | G | C | 1.08 | 0.362 | -0.009 | 2.160 | 0.0525 |
| LnAbund | 2017 | G | CC | 0.11 | 0.342 | -0.918 | 1.135 | 0.9976 |
| LnAbund | 2017 | P | C | 1.09 | 0.362 | 0.010 | 2.178 | **0.0473** |
| LnAbund | 2017 | P | CC | 0.13 | 0.342 | -0.900 | 1.153 | 0.9956 |
| LnAbund | 2017 | P | G | 0.02 | 0.342 | -1.008 | 1.045 | 1.0000 |
| LnAbund | 2017 | R | C | 1.33 | 0.362 | 0.250 | 2.418 | **0.0115** |
| LnAbund | 2017 | R | CC | 0.37 | 0.342 | -0.660 | 1.393 | 0.8179 |
| LnAbund | 2017 | R | G | 0.26 | 0.342 | -0.768 | 1.285 | 0.9400 |
| LnAbund | 2017 | R | P | 0.24 | 0.342 | -0.786 | 1.266 | 0.9535 |
| LnAbund | 2018 | CC | C | 0.03 | 0.247 | -0.710 | 0.770 | 0.9999 |
| LnAbund | 2018 | CC | G | 0.38 | 0.247 | -0.361 | 1.118 | 0.5559 |
| LnAbund | 2018 | G | C | -0.35 | 0.247 | -1.088 | 0.391 | 0.6292 |
| LnAbund | 2018 | P | C | 0.08 | 0.247 | -0.656 | 0.823 | 0.9970 |

**Table S2. cont.**

| **Response** | **Year** | **Level1** | **Level2** | **Difference** | **Std Err Dif** | **Lower CL** | **Upper CL** | **p-Value** |
| --- | --- | --- | --- | --- | --- | --- | --- | --- |
| LnAbund | 2018 | P | CC | 0.05 | 0.247 | -0.686 | 0.793 | 0.9995 |
| LnAbund | 2018 | P | G | 0.43 | 0.247 | -0.308 | 1.171 | 0.4302 |
| LnAbund | 2018 | R | C | 0.50 | 0.247 | -0.245 | 1.235 | 0.3006 |
| LnAbund | 2018 | R | CC | 0.47 | 0.247 | -0.275 | 1.205 | 0.3587 |
| LnAbund | 2018 | R | G | 0.84 | 0.247 | 0.104 | 1.583 | **0.0207** |
| LnAbund | 2018 | R | P | 0.41 | 0.247 | -0.328 | 1.151 | 0.4761 |
| SpNumb | 2014 | CC | C | -1.00 | 0.740 | -3.215 | 1.215 | 0.6640 |
| SpNumb | 2014 | G | C | -1.00 | 0.740 | -3.215 | 1.215 | 0.6640 |
| SpNumb | 2014 | G | CC | 0.00 | 0.740 | -2.215 | 2.215 | 1.0000 |
| SpNumb | 2014 | P | C | -0.17 | 0.740 | -2.381 | 2.048 | 0.9994 |
| SpNumb | 2014 | P | CC | 0.83 | 0.740 | -1.381 | 3.048 | 0.7912 |
| SpNumb | 2014 | P | G | 0.83 | 0.740 | -1.381 | 3.048 | 0.7912 |
| SpNumb | 2014 | P | R | 0.67 | 0.740 | -1.548 | 2.881 | 0.8932 |
| SpNumb | 2014 | R | C | -0.83 | 0.740 | -3.048 | 1.381 | 0.7912 |
| SpNumb | 2014 | R | CC | 0.17 | 0.740 | -2.048 | 2.381 | 0.9994 |
| SpNumb | 2014 | R | G | 0.17 | 0.740 | -2.048 | 2.381 | 0.9994 |
| SpNumb | 2015 | CC | C | 1.00 | 0.676 | -1.022 | 3.022 | 0.5864 |
| SpNumb | 2015 | CC | G | 0.67 | 0.676 | -1.355 | 2.689 | 0.8582 |
| SpNumb | 2015 | CC | P | 0.17 | 0.676 | -1.855 | 2.189 | 0.9991 |
| SpNumb | 2015 | G | C | 0.33 | 0.676 | -1.689 | 2.355 | 0.9871 |
| SpNumb | 2015 | P | C | 0.83 | 0.676 | -1.189 | 2.855 | 0.7328 |
| SpNumb | 2015 | P | G | 0.50 | 0.676 | -1.522 | 2.522 | 0.9444 |
| SpNumb | 2015 | R | C | 1.50 | 0.676 | -0.522 | 3.522 | 0.2129 |
| SpNumb | 2015 | R | CC | 0.50 | 0.676 | -1.522 | 2.522 | 0.9444 |
| SpNumb | 2015 | R | G | 1.17 | 0.676 | -0.855 | 3.189 | 0.4412 |
| SpNumb | 2015 | R | P | 0.67 | 0.676 | -1.355 | 2.689 | 0.8582 |
| SpNumb | 2016 | CC | C | 2.83 | 0.955 | -0.025 | 5.691 | 0.0527 |
| SpNumb | 2016 | CC | G | 0.50 | 0.955 | -2.358 | 3.358 | 0.9839 |
| SpNumb | 2016 | CC | P | 0.67 | 0.955 | -2.191 | 3.525 | 0.9546 |
| SpNumb | 2016 | CC | R | 0.17 | 0.955 | -2.691 | 3.025 | 0.9998 |
| SpNumb | 2016 | G | C | 2.33 | 0.955 | -0.525 | 5.191 | 0.1446 |
| SpNumb | 2016 | G | P | 0.17 | 0.955 | -2.691 | 3.025 | 0.9998 |
| SpNumb | 2016 | P | C | 2.17 | 0.955 | -0.691 | 5.025 | 0.1962 |
| SpNumb | 2016 | R | C | 2.67 | 0.955 | -0.191 | 5.525 | 0.0748 |
| SpNumb | 2016 | R | G | 0.33 | 0.955 | -2.525 | 3.191 | 0.9965 |
| SpNumb | 2016 | R | P | 0.50 | 0.955 | -2.358 | 3.358 | 0.9839 |
| SpNumb | 2017 | CC | C | 2.20 | 1.387 | -1.959 | 6.357 | 0.5230 |
| SpNumb | 2017 | CC | G | 0.50 | 1.309 | -3.432 | 4.432 | 0.9951 |
| SpNumb | 2017 | G | C | 1.70 | 1.387 | -2.459 | 5.857 | 0.7373 |
| SpNumb | 2017 | P | C | 2.20 | 1.387 | -1.959 | 6.357 | 0.5230 |
| SpNumb | 2017 | P | CC | 0.00 | 1.309 | -3.932 | 3.932 | 1.0000 |
| SpNumb | 2017 | P | G | 0.50 | 1.309 | -3.432 | 4.432 | 0.9951 |
| SpNumb | 2017 | R | C | 2.20 | 1.387 | -1.959 | 6.357 | 0.5230 |
| SpNumb | 2017 | R | CC | 0.00 | 1.309 | -3.932 | 3.932 | 1.0000 |
| SpNumb | 2017 | R | G | 0.50 | 1.309 | -3.432 | 4.432 | 0.9951 |
| SpNumb | 2017 | R | P | 0.00 | 1.309 | -3.932 | 3.932 | 1.0000 |
| SpNumb | 2018 | CC | C | 3.33 | 0.957 | 0.470 | 6.197 | **0.0177** |
| SpNumb | 2018 | CC | G | 2.67 | 0.957 | -0.197 | 5.530 | 0.0755 |
| SpNumb | 2018 | CC | P | 2.17 | 0.957 | -0.697 | 5.030 | 0.1976 |

**Table S2. cont.**

| **Response** | **Year** | **Level1** | **Level2** | **Difference** | **Std Err Dif** | **Lower CL** | **Upper CL** | **p-Value** |
| --- | --- | --- | --- | --- | --- | --- | --- | --- |
| SpNumb | 2018 | CC | R | 1.00 | 0.957 | -1.863 | 3.863 | 0.8315 |
| SpNumb | 2018 | G | C | 0.67 | 0.957 | -2.197 | 3.530 | 0.9548 |
| SpNumb | 2018 | P | C | 1.17 | 0.957 | -1.697 | 4.030 | 0.7406 |
| SpNumb | 2018 | P | G | 0.50 | 0.957 | -2.363 | 3.363 | 0.9840 |
| SpNumb | 2018 | R | C | 2.33 | 0.957 | -0.530 | 5.197 | 0.1458 |
| SpNumb | 2018 | R | G | 1.67 | 0.957 | -1.197 | 4.530 | 0.4326 |
| SpNumb | 2018 | R | P | 1.17 | 0.957 | -1.697 | 4.030 | 0.7406 |

**Table S3.** Mean and range of environmental variables used in constrained ordination analysis to explain species responses to environmental gradients. Values are Mean(Range). The mean values were used in the statistical analysis. Variables are described in Table 2 and Materials and methods section. Variable abbreviations: PAR_mean: Mean of photosynthetically active radiation, CanopyOpen – Canopy openness in percentage, Cover – Cover of understory vegetation in percentage, Ts_max – Maxima of soil temperature, SWC_mean – Mean of soil moisture, Litt_W_s – Litter weight. See Materials and methods for definition and measurement of variables.

| **Variable** | **Treatment** | **2014** | **2015** | **2016** | **2017** | **2018** |
| --- | --- | --- | --- | --- | --- | --- |
| CanopyOp | Control | 10.18 (8.06 - 14.30) | 6.54 (2.34 - 13.26) | 4.83 (3.25 - 9.50) | 2.38 (0.50 - 4.50) | 2.54 (0.75 - 3.75) |
| CanopyOp | Clear-cut | 12.09 (7.02 - 15.60) | 97.54 (94.12 - 99.84) | 89.00 (78.75 - 93.25) | 86.96 (83.50 - 91.00) | 82.92 (63.50 - 92.75) |
| CanopyOp | Gap | 11.61 (7.02 - 16.90) | 55.16 (44.46 - 73.06) | 41.58 (24.25 - 63.75) | 33.79 (18.00 - 49.50) | 25.50 (14.50 - 49.75) |
| CanopyOp | Preparation | 10.62 (7.80 - 19.24) | 29.81 (21.32 - 39.00) | 22.51 (14.82 - 41.75) | 15.41 (11.44 - 26.50) | 15.23 (9.10 - 19.75) |
| CanopyOp | Retention | 11.31 (6.24 - 13.78) | 18.11 (5.46 - 31.46) | 12.25 (4.25 - 19.75) | 11.04 (4.50 - 19.50) | 9.25 (4.75 - 16.50) |
| Cover | Control | 35.48 (18.51 - 50.32) | 49.15 (37.91 - 71.04) | 55.80 (44.50 - 71.76) | 64.44 (44.71 - 77.00) | 55.34 (35.31 - 77.90) |
| Cover | Clear-cut | 57.55 (31.02 - 90.00) | 78.25 (52.03 - 99.01) | 129.44 (115.80 - 149.70) | 139.94 (88.00 - 168.60) | 138.41 (125.00 - 165.70) |
| Cover | Gap | 43.92 (18.73 - 79.02) | 82.36 (37.60 - 141.00) | 129.79 (92.20 - 167.00) | 124.15 (104.30 - 138.00) | 123.26 (98.50 - 132.51) |
| Cover | Preparation | 59.98 (24.41 - 112.71) | 77.70 (27.63 - 127.70) | 106.60 (60.00 - 145.70) | 126.06 (74.32 - 151.10) | 117.72 (84.91 - 142.21) |
| Cover | Retention | 48.04 (13.90 - 100.11) | 57.36 (12.30 - 113.00) | 69.58 (39.91 - 107.10) | 76.50 (51.53 - 121.90) | 76.93 (47.43 - 109.11) |
| Litt_W_s | Control | 813.76 (552.25 - 988.72) | 775.50 (664.94 - 1065.17) | 780.31 (465.72 - 1069.14) | 1023.78 (855.72 - 1260.67) | 885.25 (649.81 - 1263.97) |
| Litt_W_s | Clear-cut | 804.51 (528.58 - 1103.75) | 867.18 (472.69 - 1338.42) | 216.74 (33.67 - 392.92) | 176.75 (85.17 - 338.67) | 224.07 (20.92 - 466.67) |
| Litt_W_s | Gap | 844.51 (662.92 - 1068.64) | 929.39 (617.44 - 1375.81) | 525.55 (296.36 - 1265.69) | 511.94 (430.72 - 691.28) | 451.77 (254.56 - 809.22) |
| Litt_W_s | Preparation | 858.19 (542.36 - 1075.56) | 982.13 (555.19 - 1743.14) | 512.72 (237.86 - 873.89) | 802.51 (620.64 - 1131.28) | 672.18 (341.06 - 1270.89) |
| Litt_W_s | Retention | 902.09 (569.06 - 1269.89) | 897.50 (467.00 - 1474.97) | 582.65 (346.67 - 984.69) | 962.35 (564.33 - 1721.22) | 769.07 (483.58 - 1090.25) |
| PAR_mean | Control | 21.87 (16.36 - 29.45) | 36.24 (25.81 - 50.65) | 25.35 (20.03 - 37.05) | 19.61 (11.95 - 27.64) | 24.59 (11.10 - 34.65) |
| PAR_mean | Clear-cut | 28.94 (15.11 - 40.07) | 315.87 (298.75 - 348.03) | 368.31 (332.95 - 407.05) | 333.95 (298.52 - 365.57) | 339.42 (194.72 - 426.58) |
| PAR_mean | Gap | 23.56 (15.58 - 45.43) | 147.17 (111.89 - 182.17) | 144.93 (106.45 - 193.29) | 116.74 (83.34 - 158.80) | 111.25 (40.49 - 212.33) |
| PAR_mean | Preparation | 26.18 (16.82 - 48.93) | 110.63 (92.05 - 140.36) | 91.82 (65.32 - 138.08) | 70.30 (55.58 - 95.80) | 74.15 (58.74 - 95.19) |
| PAR_mean | Retention | 26.70 (18.43 - 37.96) | 110.73 (72.86 - 144.70) | 89.90 (54.68 - 127.66) | 68.85 (43.23 - 121.31) | 64.93 (38.46 - 117.58) |
| RH_max | Control | 88.35 (86.33 - 90.20) | 91.53 (90.29 - 92.01) | 87.46 (86.54 - 88.69) | 91.59 (90.20 - 92.91) | 85.69 (83.91 - 87.81) |
| RH_max | Clear-cut | 88.54 (87.08 - 90.74) | 91.88 (90.10 - 93.06) | 87.81 (86.96 - 89.88) | 92.26 (89.53 - 94.64) | 88.70 (85.36 - 93.10) |
| RH_max | Gap | 88.61 (86.83 - 90.49) | 91.71 (90.97 - 93.01) | 87.62 (84.63 - 89.75) | 91.64 (90.58 - 93.31) | 86.92 (85.61 - 88.54) |
| RH_max | Preparation | 86.31 (74.45 - 90.09) | 91.94 (91.45 - 92.89) | 87.85 (86.83 - 89.04) | 91.58 (89.30 - 93.36) | 86.12 (84.51 - 87.90) |
| RH_max | Retention | 86.67 (75.80 - 90.39) | 91.50 (90.73 - 92.18) | 86.77 (86.29 - 87.78) | 91.28 (89.74 - 93.67) | 85.82 (83.81 - 90.33) |

**Table S3. cont.**

| **Variable** | **Treatment** | **2014** | **2015** | **2016** | **2017** | **2018** |
| --- | --- | --- | --- | --- | --- | --- |
| SWC_mean | Control | 0.27 (0.21 - 0.30) | 0.26 (0.22 - 0.30) | 0.27 (0.21 - 0.33) | 0.24 (0.20 - 0.28) | 0.19 (0.11 - 0.27) |
| SWC_mean | Clear-cut | 0.27 (0.24 - 0.31) | 0.28 (0.27 - 0.30) | 0.32 (0.29 - 0.35) | 0.30 (0.28 - 0.32) | 0.28 (0.25 - 0.31) |
| SWC_mean | Gap | 0.28 (0.24 - 0.33) | 0.33 (0.29 - 0.37) | 0.36 (0.30 - 0.41) | 0.34 (0.28 - 0.40) | 0.32 (0.26 - 0.36) |
| SWC_mean | Preparation | 0.25 (0.19 - 0.28) | 0.26 (0.18 - 0.28) | 0.27 (0.20 - 0.30) | 0.24 (0.16 - 0.28) | 0.20 (0.13 - 0.22) |
| SWC_mean | Retention | 0.26 (0.23 - 0.29) | 0.25 (0.22 - 0.30) | 0.25 (0.21 - 0.29) | 0.23 (0.20 - 0.27) | 0.19 (0.13 - 0.24) |
| T_max | Control | 17.77 (17.43 - 18.05) | 17.64 (17.13 - 18.57) | 16.86 (16.23 - 17.81) | 18.11 (17.75 - 18.37) | 20.74 (20.02 - 21.96) |
| T_max | Clear-cut | 18.10 (17.58 - 19.42) | 19.27 (18.48 - 20.32) | 19.06 (18.42 - 20.04) | 20.63 (19.73 - 21.60) | 22.31 (13.58 - 25.41) |
| T_max | Gap | 17.81 (17.48 - 18.07) | 18.49 (18.04 - 19.62) | 17.99 (17.60 - 19.53) | 19.40 (18.69 - 21.48) | 20.52 (19.55 - 21.47) |
| T_max | Preparation | 17.77 (17.14 - 18.14) | 18.39 (17.92 - 18.88) | 17.47 (17.09 - 17.86) | 19.04 (17.78 - 20.27) | 21.79 (20.81 - 23.15) |
| T_max | Retention | 17.77 (17.48 - 17.92) | 18.90 (18.07 - 20.60) | 17.65 (17.33 - 18.57) | 19.19 (18.04 - 20.32) | 21.38 (19.24 - 22.56) |
| TS_max | Control | 14.10 (13.59 - 14.40) | 14.85 (13.91 - 15.95) | 14.17 (13.67 - 15.01) | 14.63 (14.06 - 15.14) | 16.30 (15.29 - 17.19) |
| TS_max | Clear-cut | 14.56 (14.13 - 15.16) | 20.37 (17.93 - 22.15) | 18.36 (17.04 - 19.85) | 18.30 (15.15 - 20.69) | 17.96 (10.98 - 21.48) |
| TS_max | Gap | 14.00 (13.77 - 14.61) | 17.09 (15.47 - 18.82) | 15.27 (13.70 - 16.43) | 15.67 (14.48 - 17.35) | 15.86 (14.41 - 17.74) |
| TS_max | Preparation | 14.28 (13.55 - 15.05) | 17.81 (16.71 - 19.56) | 16.12 (15.51 - 16.81) | 16.42 (14.97 - 17.18) | 18.84 (17.77 - 22.20) |
| TS_max | Retention | 13.91 (13.63 - 14.09) | 18.16 (15.79 - 20.53) | 16.26 (14.87 - 17.73) | 16.91 (15.59 - 19.14) | 18.39 (15.37 - 21.59) |

**Table S4.** Marginal and conditional effects of the studied environmental variables within the RDA model. Marginal effect means the separately explained variances of the variables, while conditional effect means their explained variances within the full model. The significance of the variables were tested by Monte-Carlo simulation based pesudo F statistics based on 9999 pemutations. Variable abbreviations: PAR_mean: Mean of photosynthetically active radiation, CanopyOpen – Canopy openness in percentage, Cover – Cover of understory vegtation in percentage, Ts_max – Maxima of soil temperature, SWC_mean – Mean of soil moisture, Litt_W_s- Litter weight. See Materials and methods for definition and measurement of variables. Significant variables are in bold.

| **Marginal effects** | | | | |
| --- | --- | --- | --- | --- |
| **Variable** | **Explains %** | **pseudo-F** | **P** | **P(adj)** |
| **PAR_mean** | **4.8** | **7.0** | **0.001** | **0.0015** |
| **CanopyOpen** | **4.8** | **7.0** | **0.001** | **0.0015** |
| **Cover** | **4.1** | **5.9** | **0.001** | **0.0015** |
| **Ts_max** | **3.3** | **4.8** | **0.001** | **0.0015** |
| **SWC_mean** | **3.3** | **4.7** | **0.002** | **0.0024** |
| **Litt_W_s** | **1.7** | **2.5** | **0.016** | **0.016** |
|  |  |  |  |  |
| **Conditional effects** | | | | |
| **Variable** | **Explains %** | **pseudo-F** | **P** | **P(adj)** |
| **PAR_mean** | **4.82** | **7.0** | **0.001** | **0.003** |
| **SWC_mean** | **2.67** | **4.0** | **0.001** | **0.003** |
| **Cover** | **1.77** | **2.7** | **0.001** | **0.024** |
| CanopyOpen | 1.11 | 1.7 | 0.001 | 0.168 |
| Ts_max | 0.71 | 1.1 | 0.001 | 0.442 |
| Litt_W_s | 0.25 | 0.4 | 0.001 | 0.923 |
